# Valorization of Wine Lees in the Production of Reduced-Lipid Nutritive Muffins

**DOI:** 10.3390/foods15122113

**Published:** 2026-06-11

**Authors:** Aurica Chirsanova, Alina Boiștean, Xenia Pascari, Rodica Siminiuc, Ecaterina Gore

**Affiliations:** 1 Department of Food and Nutrition, Faculty of Food Technology, Technical University of Moldova, 9/9 Studentilor St., MD-2045 Chisinau, Moldova; alina.boistean@toap.utm.md (A.B.); rodica.siminiuc@adm.utm.md (R.S.); 2 Reference Centre for Food and Feed Analysis, German Federal Institute for Risk Assessment (BfR), Max-Dohrn-Straße 8-10, 10589 Berlin, Germany; xenia.pascari@bfr.bund.de; 3 Laboratoire URCOM, Université Le Havre Normandie, 25 Rue Philippe Lebon, BP 1123, 76063 Le Havre, France; ecaterina.gore@univ-lehavre.fr

**Keywords:** residual wine yeast, fat replacement, nutritional enhancement, sustainable bakery products

## Abstract

The valorization of winemaking by-products is a sustainable strategy consistent with circular bioeconomy principles and current public health priorities. This study aimed to evaluate residual oenological yeast sediment from local Moldovan grape varieties, Viorica and Fetească Regală, as a multifunctional ingredient and partial fat replacer in muffins. Sunflower oil was replaced with wine lees (WL) at 20%, 35%, and 50%, and the obtained products were analyzed in terms of physicochemical, nutritional, microbiological, colorimetric, and sensory characteristics. WL incorporation reduced the caloric value by up to 10% and decreased lipid content, while contributing to higher protein and dietary fiber levels. Moisture values remained within acceptable limits, whereas titratable acidity increased with the substitution level (*p* < 0.05). Muffin density showed a slight increase, and water absorption capacity improved markedly, reaching 269%, mainly due to the fiber-rich composition of WL. Color analysis indicated reduced lightness and increased redness, associated with yeast pigments and thermal reactions during baking. Microbiological results showed lower total viable counts with increasing WL addition; however, the 50% substitution level exceeded the permissible limits for yeasts and molds. Sensory evaluation indicated that the muffin with 20% WL was the most acceptable sample. Overall, WL may be considered a promising sustainable ingredient for developing reduced-fat muffins with improved nutritional value.

## 1. Introduction

In many Western countries, average fat intake typically ranges between 35 and 40% of total energy intake, exceeding WHO recommendations [1] which has been associated with an increased risk of obesity, cardiovascular diseases and metabolic disorders, hence the continued public health emphasis on reducing total and saturated fat consumption [2]. Partial replacement of lipids with yeast-derived β-glucans is a promising strategy to reduce fat content while increasing dietary fiber, provided that technological and sensory functions of lipids are carefully compensated [3].

Viticulture and winemaking represent major sectors of the global agri-food economy [4]. According to the International Organisation of Vine and Wine, the global vineyard surface area reached approximately 7.1 million ha in 2024, while world wine production was estimated at 225.8 million hL [5]. One of the most abundant by-products generated during vinification is wine lees (WL), a sediment composed primarily of yeast cells, potassium and calcium tartrate crystals, polysaccharides, polyphenols, proteins, and mineral matter. Although traditionally regarded as waste, WL have recently attracted significant scientific interest due to their promising nutritional and technological potential.

The physicochemical properties of WL vary according to grape variety, fermentation conditions, and wine type (white, rosé, or red). Studies have reported pH values ranging from 3.38 to 3.45 for WL derived from different wine styles, with acidity largely associated with precipitated tartrate salts formed during alcoholic fermentation [6]. WL also contain substantial antioxidant capacity, total nitrogen, crude fat, and ash, with red WL typically exhibiting higher phenolic and anthocyanin levels compared to WL from white wines [7].

Mineral composition further distinguishes the lees of various wine types. White WL contain higher amounts of Ca and K, rosé lees are richer in P, red WL exhibit elevated levels of Mg, Fe, and transition metals such as Zn and Pb [8]. These differences reflect both grape physiology, and winemaking parameters, including fermentation temperature, pH, maceration, and alcohol content.

From a nutritional standpoint, WL constitute a rich source of carbohydrates, nitrogenous compounds, vitamins, essential amino acids, lipids, and dietary fibers. Yeast proteins exhibit high biological value; however, due to their intracellular localization, cell-wall disruption through mechanical (shear force) or non-mechanical (chemical) methods is required to increase digestibility and bioavailability [9]. Studies conducted at the Institute of Microbiology and Biotechnology of the Academy of Sciences of Moldova demonstrated that essential amino acids such as arginine, leucine, lysine, threonine, and isoleucine are present in significant amounts in WL from both white and red grapes, making them a valuable source of essential amino acids [10].

WL also serve as a natural source of lipids, including sterol esters, triglycerides, free fatty acids, mono- and diglycerides, and phospholipids [11]. These lipids play important roles in cellular membrane structure, emulsification, and the stabilization of food emulsions. Fractionation studies reveal that sterol esters and waxes represent over 40% of total lipids in white WL, followed by triglycerides (18–20%) and sterols (13%) [12]. Fatty acids such as palmitoleic, oleic, and myristic acids dominate the lipid profile and contribute to functional characteristics important in food formulations.

Dietary fibers in WL include both insoluble (cellulose, hemicellulose, lignin) and soluble fractions, particularly β-glucans. Insoluble fibers support gut health by improving intestinal transit, whereas soluble β-glucans exhibit viscosity, water-holding capacity, and emulsifying properties, enabling their application as natural thickeners and texture modifiers in food systems [13]. β-Glucans also demonstrate prebiotic, hypocholesterolemic, and immunomodulatory effects, further enhancing the functional value of WL [14]. Moreover, WL possess natural antioxidant and antimicrobial activities. Their application as a natural preservative in meat products has shown reductions in pH, color variations, lipid oxidation, and microbial load, while enhancing phenolic content and sensory attributes such as bakery-like and raisin-like notes [15].

In recent years, research shows that flour and fermentation media substitution with WL have been successful in improving the nutritional profiles of these products for a broader application in foods [15,16].

These examples illustrate a growing trend toward valorizing winery by-products within the framework of circular bioeconomy [17]. Taken together, these findings highlight WL as a multifunctional and sustainable ingredient rich in proteins, essential amino acids, lipids, minerals, β-glucans, and manoproteins. Their valorization aligns with principles of circular bioeconomy and offers substantial potential for use in bakery [15], dairy [18], meat [12], and functional food products [19]. Given their abundance and diverse biochemical composition, WL represent an under-utilized resource that can significantly enhance nutritional quality and technological performance in novel food formulations.

Muffins are widely consumed bakery products; however, their conventional formulations often contain considerable amounts of fat, which contributes to increased energy value. Therefore, reducing lipid content in muffins while preserving acceptable technological and sensory properties remains an important formulation challenge. In this context, wine lees may represent a promising ingredient for partial fat replacement due to their dietary fiber, protein, and bioactive compound content. However, limited information is available on the use of wine lees from local Moldovan grape varieties as partial fat replacers in muffin formulations, particularly regarding product quality, microbiological safety, and sensory acceptance.

Therefore, the aim of this research was to evaluate the technological, nutritional, microbiological, colorimetric, and sensory effects of partially replacing sunflower oil with WL from Viorica and Fetească Regală varieties in muffin formulations, to develop reduced-fat bakery products, while promoting waste recovery and sustainable ingredient use.

## 2. Materials and Methods

### 2.1. Reagents

All chemicals and reagents used in the analyses were of analytical grade, purchased from the specialized supplier Ecochimie SRL (Chişinău, Republic of Moldova), and accompanied by quality certificates.

### 2.2. Materials and Sample Formulation

In this research, WL (*Saccharomyces cerevisiae*) obtained during the alcoholic fermentation of must from the local grape varieties Viorica and Fetească Regală were used. The WL, supplied by Purcari Winery (Stefan-Voda, Republic of Moldova), were collected at the end of the alcoholic fermentation process, after wine separation, and stored under controlled conditions (4 ± 1 °C) until their use in muffin formulation. Before incorporation into muffin formulations, the WL were characterized for basic physicochemical and nutritional parameters, including pH, dry matter, ash, carbohydrates, lipids, proteins, β-glucans, and fractional lipid composition. Table 1 presents the technological formulation of the muffins, designed with a minimal number of ingredients to correctly assess the effect of WL on the product’s properties. A simplified recipe was applied to avoid the influence of additional components and to maintain cost efficiency, while ensuring that the functional impact of the sediment could be accurately evaluated. All ingredients used in the formulation, except for the WL, were commercially available and purchased from local retail sources.

The muffins were prepared as follows: the dry ingredients (wheat flour, salt, sugar, and baking powder) were placed in a planetary mixer (model Plutone 7, Sirman, Italy) and blended at medium speed for 5 min, together with drinking water, sunflower oil, and eggs, to obtain the control sample, designated as M0% (Table 1). In the experimental formulations, sunflower oil was partially replaced with WL substitution levels of 20%, 35%, and 50% of the total oil weight. These samples were further coded according to the origin of the WL: V for Viorica (MV20%, MV35%, MV50%) and FR for Fetească Regală (MFR20%, MFR35%, MFR50%). The resulting batters were portioned into baking molds and baked in a ventilated oven (UX-XFT193-ROSSELLA, Unox, Cadoneghe, Italy) at 180 ± 2 °C for 20 min. Each formulation consisted of a single batch of muffin batter (≈263 g total mass) and was prepared in triplicate.

### 2.3. Physicochemical Analysis of Muffins

#### 2.3.1. Moisture Content

The water content of the muffins was determined in triplicate for each formulation. The samples were ground in a mill, and portions of 3 g were transferred to pre-weighed moisture dishes and placed in a laboratory dryer (SLW series, POL-EKO, Warsaw, Poland) at 103 ± 2 °C until a constant mass was reached. The water content was calculated as the difference in weight before and after drying, expressed as a percentage [20].

#### 2.3.2. Total Titratable Acidity

The determination of total titratable acidity (TTA) of muffin samples was performed in triplicate according to the procedure described by Dingeo et al. [21]. Briefly, muffin samples were homogenized, and an accurately weighed portion (10 g) was suspended in 100 mL of distilled water. The mixture was filtered using Whatman filter paper (No. 1), and the filtrate was subjected to titration with 0.1 M NaOH solution, using phenolphthalein as an indicator. The titration was carried out until a persistent faint pink coloration was observed, corresponding to a pH of approximately 8.1. The results were expressed as the percentage of lactic acid equivalents (% *w*/*w*), calculated from the volume of NaOH consumed.

#### 2.3.3. Volume and Crumb Density

Specific volume was calculated by dividing the volume by the weight. The volume (mL) was determined using the displacement of millet seeds, while the weight (g) was measured using an analytical balance [22]. The analysis was performed in triplicate.

#### 2.3.4. Water Absorption Characteristics (WAC)

Water absorption refers to the ability of a material to absorb water when immersed in it and is represented with water absorption capacity. Water Absorption Index (WAI) is defined as the ratio of water absorbed by a material in a saturated state over the weight of dry matter. WAC was determined by an immersion method using a stainless-steel mesh chamber, based on the mass difference before and after soaking at 20 °C, as described by Khaleel et al. [23]. The chamber was then submerged in a container filled with water at 20 °C for 6 min. After immersion, the chamber was removed and held at an inclined position for 30 s to allow excess water to drain. The chamber was then wiped on the outside and weighed first with the wet sample, then empty (after removing the sample). The analysis was performed in triplicate. The WAC, X (%), is calculated using the following formula:

(1) $$X=\frac{\left(m-m_{1}\right)}{\left(m_{2}-m_{1}\right)}\;\times \;100$$

where *m*—mass of the chamber with the wet sample (g);

*m*_1_—mass of the empty chamber (after immersion and drying the exterior) (g);
*m*_2_—mass of the chamber with the dry sample (g).

#### 2.3.5. Microbiological Analysis

Total viable counts were determined using the pour plate method based on serial decimal dilutions. An accurately weighed portion of 10 g of muffin sample was aseptically homogenized with 90 mL of sterile water to obtain the initial suspension (10^−1^).

One milliliter of the appropriate dilution was transferred into sterile Petri dishes, followed by the addition of molten culture medium. Mueller–Hinton Agar (Oxoid, Thermo Fisher Scientific, London, UK) was used for the enumeration of total aerobic mesophilic microorganisms, while Sabouraud Dextrose Agar (Oxoid, Thermo Fisher Scientific, UK) was employed for the determination of yeasts and molds, due to its suitability for fungal growth. The culture media were prepared according to the manufacturers’ instructions.

After gentle rotation to ensure uniform distribution of the inoculum, the plates were allowed to solidify, inverted, and incubated at 30 ± 1 °C for 72 ± 3 h under aerobic conditions. The results were expressed as colony-forming units per gram of sample (CFU/g). The analysis was performed in triplicate.

#### 2.3.6. Color Assessment of WL and Muffins

The color was evaluated based on CieLab principles, described by Covaliov et al., using the Konica Minolta colorimeter CR-400 (Osaka, Japan) [24]. Color analysis was performed for both WL and muffins, at the level of the crust and the crumb. Color differences were recorded in the CIE L*a*b* scale, in terms of lightness (L*) and color (a*—redness, b*—yellowness). Chroma (C*) and hue (h°) were also recorded. Furthermore, the total color difference (ΔE) was calculated using the following formula:

(2) $$∆E=\sqrt{(∆}{L^{*})}^{2}+({{∆a}^{*})}^{2}+({{∆b}^{*})}^{2},$$

where ΔL*, Δa*, and Δb* represent the differences in lightness, redness/greenness, and yellowness/blueness between the control and experimental samples, respectively.

#### 2.3.7. Calculation of Nutritional Value

The nutritional value of the experimental muffins was calculated based on the data for each ingredient from the database https://fdc.nal.usda.gov/ of the composition and nutritional value of food products [25]. The nutritional value of the prepared muffins was calculated based on the nutrient composition per 100 g of each raw ingredient used in the formulation. The caloric value of the muffin samples was calculated with the following equation:

(3) Calories, kcal/100 g = 9 × F + 4 × C + 4 × P,

where F, C, and P represent the total amount of fat, carbohydrates, and protein in 100 g of muffin sample.

#### 2.3.8. Sensory Analysis

A quantitative descriptive sensory analysis (QDA) was conducted in the sensory test room of the Technical University of Moldova; the QDA was designed according to ISO 6658:2017 standards [26]. Sensory evaluation was performed by a trained panel consisting of 15 trained assessors: 11 females and 4 males (mean age 32 ± 6 years), with prior experience in descriptive sensory analysis. Before the formal evaluation, the assessors participated in a short familiarization and calibration session, during which the evaluation procedure, the five-point sensory scale, and the use of sensory descriptors were discussed and harmonized. Descriptor understanding and consistency in descriptor use among assessors were checked before sample evaluation. The panelists collaboratively selected the descriptors and established their operational definitions prior to assessment. The evaluated attributes encompassed external characteristics (typical color, uniformity of bake, and surface cracking), crumb appearance (characteristic color, color uniformity, and porosity), textural properties (hardness, springiness, and gumminess), as well as odor and flavor descriptors (characteristic, oily, and foreign notes), and overall product quality. The intensity of each sensory attribute was assessed using a five-point unstructured linear scale. Test samples were randomly collected from each production batch, coded, and presented in random order in separate plastic containers. Between successive evaluations, the panelists rinsed their mouths with still drinking water to minimize carryover effects. The study was approved by the UTM Ethics Committee and conducted in accordance with GDPR regulations regarding personal data protection.

### 2.4. Physicochemical and Nutritional Composition Analysis of WL

The following physicochemical parameters in WL were determined at Institute X using standard analytical methods: the Bligh and Dyer method for fractional lipid estimation, the Anthrone method for total carbohydrates, and the Lowry method for total protein content [27].

β-Glucans were extracted from WL using an enzymatic method combined with ultrasonic treatment. The ultrasonic assistance enhances cell disruption and mass transfer, thereby improving the efficiency of enzymatic hydrolysis and increasing the yield of β-glucans [28]. A 100 g portion of the sample was adjusted to pH 10–11 using 1 N NaOH. The sample was then subjected to ultrasonic treatment at 30 kHz and 70 °C for 30 min. Subsequently, the pH was lowered to 6 using sulfuric acid (H_2_SO_4_). The sample was enzymatically treated by adding 0.2 g of Mannanase to the WL, followed by incubation in a water bath at 48 °C for 90 min. After incubation, the mixture was centrifuged at 10,000 rpm for 10 min, and the precipitate was washed repeatedly with water until the washings reached a neutral pH (6.5–7.0). The final product was dried at 50 °C for 3 days. All analysis were performed in triplicate.

### 2.5. Statistical Analysis

Statistical differences between samples were assessed using analysis of variance (ANOVA) at a significance level of α = 5%, performed with XLStat 2026.0.1 software (Lumivero, Denver, CO, USA). When significant differences were detected, means were compared using Tukey’s Honestly Significant Difference (HSD) post hoc test. Means followed by different letters were considered significantly different at *p* < 0.05. All analytical measurements were performed in triplicate (*n* = 3), and data are presented as mean ± standard deviation (SD). For sensory evaluation, data were obtained from 15 trained assessors (*n* = 15). In addition, Principal Component Analysis (PCA) was performed to explore the relationships among variables and to visualize the multivariate distribution of samples. The PCA was conducted using standardized data, and the first two principal components were retained for interpretation.

## 3. Results and Discussions

### 3.1. Characterization of WL

#### 3.1.1. Physicochemical and Nutritional Composition of WL

In this study, WL biomass obtained from two white wine varieties (Viorica and Fetească Regală) was analyzed to assess their potential for valorization as a functional ingredient. The physicochemical and nutritional parameters of the WL are presented in Table 2. These indicators provide insight into their technological relevance and allow identification of suitable application domains.

The pH values (3.19–3.21) fall within the typical range reported for white WL, confirming their acidic nature, which is characteristic of vinification residues [29].

The carbohydrate content ranged from 14.21% DM in Viorica lees to 19.41% DM in Fetească Regală lees, with minor differences between varieties. Soluble carbohydrates derived from grape-origin polysaccharides constitute an important fraction of WL and are of particular interest due to their contribution to aroma and sensory attributes [30].

Lipid content (10–11% DM) was slightly higher than the values reported by de Andrade Bulos et al. (8.5–9.2%), suggesting that white WL from these local varieties may exhibit an elevated lipid fraction while remaining within the biologically plausible range for autolyzed yeast material [31]. Elevated lipid levels indicate potential applications in nutraceutical or biotechnological processes, such as extraction of phospholipids, triglycerides, or functional lipid compounds [32].

In recent years, *Saccharomyces cerevisiae* biomass has gained increasing attention as a high-yield source of β-glucans, with recovery rates reaching up to 25% and offering both technological and economic advantages [33]. In the present study, the β-glucan content of white WL (19–21%) exceeded the values reported for most plant sources and was comparable to the highest levels documented in vinification yeasts.

#### 3.1.2. Fractional Lipid Composition of WL

Lipid composition plays a critical role in fermentation dynamics. High lipid levels in grape musts may enhance the synthesis of higher alcohols and modulate ester formation, thereby influencing the sensory profile of white wines [34]. Triglycerides represented the dominant lipid class (58–60%), followed by phospholipids (11–12%) and esters (7–9%), presented in Table 2. Previous research has shown that WL from red varieties contain much higher levels of esters—34.91% in Fetească Neagră, 24.22% in Rară Neagră, and 23.22% in homemade wines—highlighting a strong varietal influence on lipid fractionation [27].

Triglyceride levels around 60% are consistent with findings in brewing yeasts, where triglycerides represent the main storage lipids [35]. According to Fornairon-Bonnefond et al. (2002), the total lipid content of S. cerevisiae lees typically ranges from 3% to 15% of dry mass and consists of triglycerides, membrane phospholipids, and sterols [36].

Pueyo Pérez et al. (2000) also observed the release of multiple lipid classes during yeast autolysis in a model wine system, including triacylglycerols, 1,3-diacylglycerols, monoacylglycerols, free fatty acids, sterol esters, and sterols [37]. Their data indicate dynamic lipid release in the early stages of autolysis, although phospholipids were not detected in autolysates.

Because the lipid profile of white WL remains insufficiently documented in the literature, meaningful comparisons are difficult. Most existing studies focus on red wines or grape musts, where lipid fraction distribution differs substantially from that of white wines.

### 3.2. Physicochemical Properties of Muffins

#### 3.2.1. Moisture and Dry Matter Content

**Figure 1:**
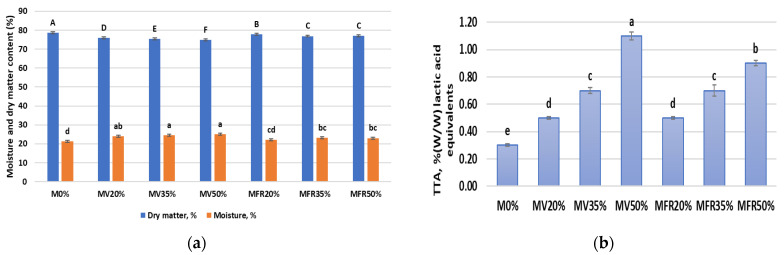
Physicochemical parameters of muffins enriched with WL: (**a**) moisture content, and dry matter, (**b**) total titratable acidity. Abbreviations used: M0%: control formulation without WL (100% sunflower oil). MV20%, MV35%, MV50%: formulations in which 20%, 35% or 50% of sunflower oil was replaced by WL originating from Viorica. MFR20%, MFR35%, MFR50%: formulations in which 20%, 35% or 50% of sunflower oil was replaced by WL originating from Fetească Regală. Means followed by different uppercase letters for moisture content and different lowercase letters for dry matter content are significantly different according to Tukey’s HSD test (*p* < 0.05). Values are expressed as mean ± SD (*n* = 3).

The dry matter content of the muffins ranged between 21 and 25%, corresponding to moisture levels of 75–79%, depending on the proportion and type of WL incorporated (Figure 1a). The addition of WL stemming from both wine varieties had a significant effect on dry matter content compared to the control formulation (*p* < 0.05). Samples enriched with Viorica WL exhibited slightly higher dry matter values compared with those containing Fetească Regală WL. A 20% substitution of sunflower oil the WL obtained from Viorica wine fermentation (MV20) significantly increased the proportion of dry matter, while higher substitution rates did not lead to further increases. In the case of Feteasca Regala WL (MFR), the significant increase in dry matter was achieved after substituting 35% and 50% of the sunflower oil.

These results fall within the range commonly reported for muffin-type bakery products and may be influenced by formulation characteristics, fat content, and the incorporation of fiber-rich ingredients [38].

#### 3.2.2. Total Titratable Acidity (TTA) of Muffins

Figure 1b presents the TTA values of muffins formulated with WL originating from Viorica and Fetească Regală grape varieties at substitution levels of 0%, 20%, 35%, and 50%. The increase in titratable acidity observed with higher WL incorporation levels was statistically significant (*p* < 0.05), confirming the direct contribution of organic acids naturally present in wine lees to the overall acidity of the muffins. In the control samples (0%), an acidity value of 0.3% *w*/*w* was recorded. At 20% substitution, acidity significantly increased to 0.5% *w*/*w* for both WL sources, maintaining comparable behavior across the formulations.

A further increase to 35% WL addition resulted in acidity values of 0.7% *w*/*w* for both Viorica and Fetească Regală, demonstrating a proportional rise in titratable acidity with the inclusion level of WL. The most pronounced differences appeared at the 50% substitution level, where muffins with Viorica WL reached 1.1% *w*/*w*, whereas those containing Fetească Regală WL exhibited a slightly lower value of 0.9% *w*/*w*. This divergence suggests a higher intrinsic acidity potential or buffer capacity of Viorica WL at elevated incorporation levels. Overall, total titratable acidity increased consistently with higher WL incorporation, reflecting the contribution of organic acids and fermentation-derived metabolites naturally present in WL.

#### 3.2.3. Determination of Muffin Density

The density of muffin samples was determined as the ratio between sample mass and volume, following the principles applied to flour confectionery products [22]. Statistically significant differences in density values (*p* < 0.05) were observed at higher substitution levels, indicating that WL addition influenced batter aeration and structural compactness.

**Figure 2:**
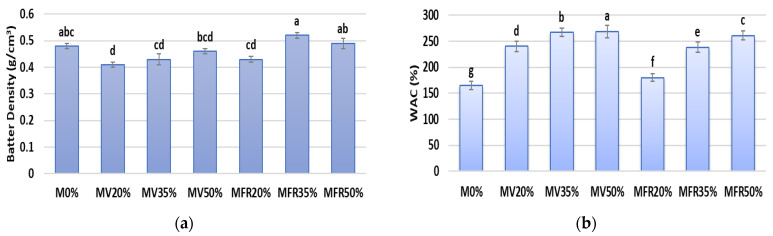
Batter density (**a**) and water absorption capacity (WAC) (**b**) of muffin formulations with varying levels of WL. Abbreviations used: M0%: control formulation without WL (100% sunflower oil). MV20%, MV35%, MV50%: formulations in which 20%, 35% or 50% of sunflower oil was replaced by WL originating from Viorica. MFR20%, MFR35%, MFR50%: formulations in which 20%, 35% or 50% of sunflower oil was replaced by WL originating from Fetească Regală. Means followed by different letters are significantly different according to Tukey’s HSD test (*p* < 0.05). Values are expressed as mean ± SD (*n* = 3).

Figure 2a illustrates the batter density which was estimated by calculating the volume via the displacement method, in which millet grains served as the volumetric indicator. The initial volume occupied by the indicator was compared with the volume remaining after immersion of the muffin sample, and the volume difference was used to calculate sample density.

A progressive decrease in muffin height was observed with increasing fat replacement, accompanied by a slight reduction in sample volume. The highest volumes were recorded for M0%, MV20%, and MFR20%, whereas MV50% and MFR50% exhibited the lowest volumes. Correspondingly, a modest increase in density was detected in the formulations containing 50% WL sediment: MV50% reached 0.52 g/cm^3^ and MFR50% reached 0.49 g/cm^3^. This increase could be attributed to the high water-binding capacity and fiber content of the WL, which reduce batter aeration and expansion during baking. Similar findings were reported in previous studies evaluating fat replacement in cakes using WL derivatives, where elevated fiber content and water adsorption led to reduced volume and slightly denser crumb structures [39].

Despite these changes, all formulations remained within the acceptable density range of 0.35–0.55 g/cm^3^ [40]. These results suggest that fat replacement with WL sediment up to 50% preserves acceptable product density and volume, while higher substitution levels may compromise structural characteristics [41].

#### 3.2.4. Water Absorption Capacity (WAC)

WAC is an important technological parameter in bakery systems, as it reflects the ability of hydrophilic groups in proteins, polysaccharides, and dietary fibers to bind and retain water within the food matrix. This characteristic directly influences batter viscosity, crumb structure, texture, and overall product stability during baking.

The WAC of the muffin samples enriched with WL is presented in Figure 2b. The control formulation (M0%) exhibited the lowest WAC value (165%), while muffins containing Viorica lees showed a progressive increase from 240.25% (MV20%) to 269.16% (MV50%). A similar trend was observed for samples enriched with Fetească Regală lees, where WAC increased from 180% (MFR20%) to 261.33% (MFR50%). These results indicate a clear dose-dependent enhancement of water absorption with increasing proportions of WL.

Enriched muffins, especially those containing 35 and 50% WL, showed a significant increase in water absorption capacity, with values reaching 269%. This effect is likely due to the high content of dietary fiber and yeast cell wall polysaccharides, particularly β-glucans and mannoproteins, which are known for their strong ability to bind and retain water through capillary and adsorption mechanisms [42].

Comparable findings were reported in studies evaluating the incorporation of unconventional raw materials into muffin formulations. For instance, pumpkin flour–enriched muffins demonstrated WAC values ranging from 164% to 184%, similarly attributed to increased levels of proteins and dietary fibers capable of enhancing water retention. Consistent with these observations, the higher WAC values recorded in muffins enriched with WL suggest that yeast-derived fibers behave analogously to plant-based fibers by promoting moisture retention and structural hydration within the crumb matrix.

#### 3.2.5. Principal Component Analysis (PCA)

**Figure 3:**
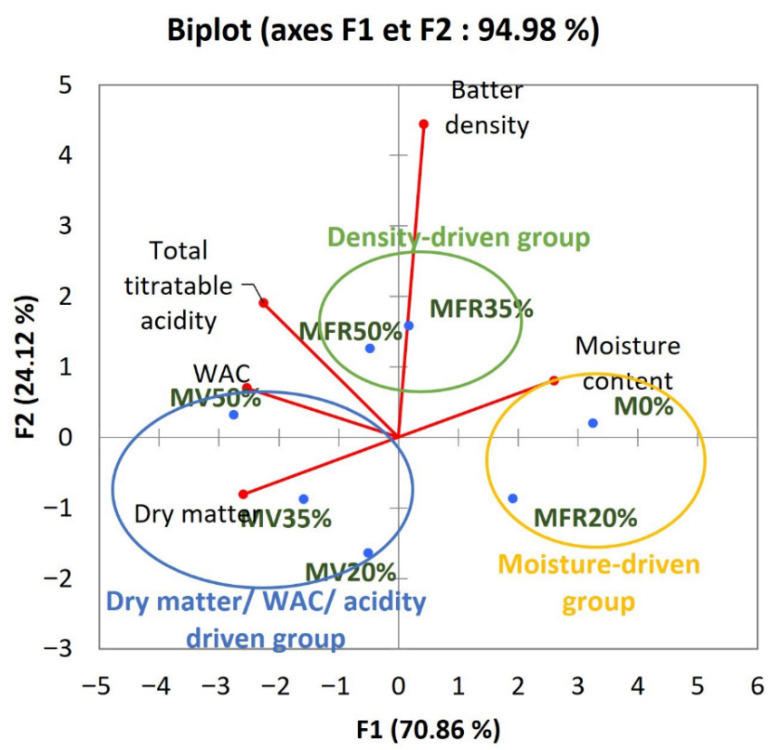
Principal component analysis (PCA) of muffin formulations enriched with wine lees (WL). Abbreviations used: M0%: control formulation without WL (100% sunflower oil). MV20%, MV35%, MV50%: formulations in which 20%, 35% or 50% of sunflower oil was replaced by WL originating from Viorica. MFR20%, MFR35%, MFR50%: formulations in which 20%, 35% or 50% of sunflower oil was replaced by WL originating from Fetească Regală.

To further elucidate the relationships among the analyzed physicochemical parameters and to visualize the clustering pattern of muffin formulations, principal component analysis (PCA) was performed, and the resulting biplot is presented in Figure 3.

Principal Component Analysis (PCA) explained 94.98% of the total variance with the first two components (F1 = 70.86%; F2 = 24.12%), indicating that the two-dimensional representation provides a highly reliable description of the data structure.

The first principal component (F1), accounting for most of the variance, clearly discriminates samples according to a moisture–dry matter gradient. Moisture content is positively associated with F1, whereas dry matter, water absorption capacity (WAC), and total titratable acidity are negatively associated. This opposition indicates a strong negative correlation between moisture and dry matter, which is consistent with their physicochemical relationship. Samples M0% and MFR20% are positioned on the positive side of F1 and are therefore associated with higher moisture content. In contrast, MV50%, MV35%, MV20%, and MFR50% are located on the negative side of F1, indicating stronger association with higher dry matter content, WAC, and TTA.

The second principal component (F2) further differentiates samples according to structural characteristics, mainly driven by batter density. Batter density loads positively on F2, distinguishing MFR35% and MFR50%, which are in the upper region of the plot and thus associated with higher density values. Conversely, samples such as MV20% and MV35% are positioned in the lower part of the axis, indicating lower association with batter density and relatively stronger contribution from dry matter.

The PCA therefore reveals three main clusters: (i) moisture-associated samples (M0%, MFR20%), (ii) dry matter/WAC/acid-associated samples (MV formulations), and (iii) density-associated samples (MFR35%, MFR50%). Overall, the results demonstrate that formulation differences primarily influence the moisture–dry matter balance, while structural properties such as batter density represent a secondary but distinct dimension of variability.

### 3.3. Proximate Composition of Muffins

The nutritional profile of the muffins prepared with different levels of vegetable oil replacement is summarized in Table 3. The substitution of oil with WL from the Viorica and Fetească Regală grape varieties resulted in measurable changes in macronutrient composition and energy value. WL contains only 1.5% lipids, compared with 99.9% lipids in vegetable oil, which considerably influenced the fat content of the final products.

The control sample (M0%) contained a total of 11.6 g lipids/100 g muffins, whereas the formulations with 50% replacement reached 7.02 g lipids/100 g muffins and 7.07 g lipids/100 g muffins for MV 50% and MFR 50%, respectively. Consequently, the energy value decreased from 1297.43 kJ/100 g in the control to 1199.47 kJ/100 g and 1198.85 kJ/100 g for the 50% replacement samples prepared with Viorica and Fetească Regală WL, respectively. This corresponds to an approximate 10% reduction in caloric density.

Protein content increased proportionally with the addition of WL, reaching 9.50 g/100 g (MV 50%) and 9.42 g/100 g (MFR 50%) compared with 5.80 g/100 g in the control. Carbohydrates remained relatively constant (46–47 g/100 g), as the amounts of flour and sugar were identical across all formulations.

Beyond macronutrient modification, the incorporation of WL likely contributed with additional micronutrients naturally present in yeast biomass, such as vitamins B_1_ and B_5_ and minerals such as Zn [43]. These micronutrients are consistent with the metabolic requirements of *Saccharomyces cerevisiae* during fermentation, as reported in previous studies [44]. Therefore, the addition of WL not only reduced the caloric content of the muffins but also enhanced their nutritional profile.

The results demonstrate that WL is an effective and sustainable fat replacer in muffin formulations, enabling caloric reduction while contributing valuable micronutrients. Such a decrease in energy density may be particularly beneficial for individuals who follow specific dietary regimens or monitor their daily caloric intake.

### 3.4. Color Analysis of Muffins

Consumers are generally attracted to a moderately darker crust in baked goods [45]. The addition of WL significantly modified the color of the muffins (Table 4), which can also be attributed to browning reactions occurring during baking, namely sugar caramelization and the Maillard reaction. The color changes observed in WL-enriched muffins may be associated with pigments derived from WL and thermal browning reactions occurring during baking; however, the specific contribution of Maillard-reactive amino acids and sugars requires further investigation [39]. Furthermore, crumb color was affected not only by the muffin ingredients but also by pigments derived from the WL, which contributed to higher color parameter values.

Color measurements were performed using a digital instrument, with five measurements taken at different points on both the crust and crumb.

The obtained results reveal clear trends associated with the increasing substitution levels (20%, 35%, and 50%) and the type of WL. Control muffins (M0) showed the highest values of lightness for both crust (54.42) and crumb (56.77), indicating a bright appearance typical of conventional baked products. With the progressive incorporation of WL, a significant decrease in L* was observed. At 50% substitution, L* dropped sharply to 41.8 in the crust and 41.8 in the crumb for MV samples, while MFR formulations reached values as low as 13.99 in the crust. This reduction suggests pronounced darkening, which is attributable to both the intrinsic pigments of the WL and possible Maillard reactions intensified by the higher content of residual compounds.

In addition to thermal browning reactions, the non-enzymatic oxidation of phenolic compounds during baking may also contribute to the formation of darker pigments, including quinone-derived compounds and melanoidin-like structures. However, this mechanism was not directly assessed in the present study and should be further investigated.

The a* values, indicative of the red–green balance, also increased with the level of substitution. In MV muffins, crust values rose from 10.52 in the control to 21.02 at 50% enrichment, reflecting a deeper reddish hue. Interestingly, MFR samples maintained moderate values, ranging from 16.37 to 18.83 in the crust, but presented negative a* values in the crumb at lower substitution levels, indicating a shift towards a greenish tint. Such differences underline the distinct color-imparting capacity of the two by-products, likely influenced by their specific polyphenolic profiles. For the b* parameter, control muffins recorded the highest values (34.73 for crust and 25.06 for crumb), consistent with the expected yellow tones of wheat-based bakery products. With sediment incorporation, a gradual decline was observed. At 50% MFR enrichment, b* values fell drastically to 12.48 for crust and 12.49 for crumb, suggesting a loss of brightness and yellow shades.

**Figure 4:**
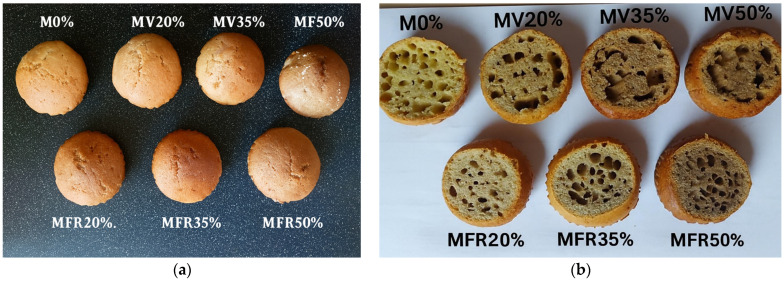
(**a**) External appearance of muffin samples with different levels of fat replacement using WL. (**b**) Cross-section of the experimental muffins. Abbreviations used: M0%: control formulation without WL (100% sunflower oil). MV20%, MV35%, MV50%: formulations in which 20%, 35% or 50% of sunflower oil was replaced by WL originating from Viorica. MFR20%, MFR35%, MFR50%: formulations in which 20%, 35% or 50% of sunflower oil was replaced by WL originating from Fetească Regală.

The calculated ΔE values highlight the perceptible differences between enriched and control samples. The highest deviations were noted at 50% addition, reaching 16.86 for MV muffins and 26.89 for MFR muffins. The data demonstrate that MFR inclusion, particularly at higher levels, exerts a stronger impact on color attributes than MV (Figure 4).

Similar observations were made by Tolve et al. and Nakov et al., who studied color changes in bread and muffins enriched with grape pomace and reported a marked decrease in L* and a shift toward red hues (increased a*), attributing these effects to interactions between polyphenolic compounds and dough matrix components; these trends are fully consistent with our present findings in muffins, in which incorporation of WL led to progressive darkening and hue shifts with rising substitution levels [46,47].

### 3.5. Sensory Analysis

**Figure 5:**
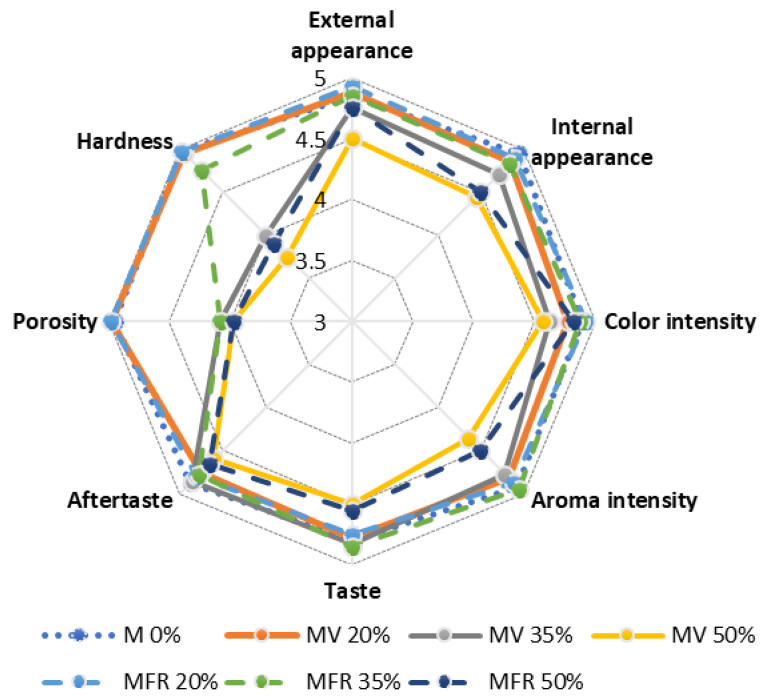
Radar plot of the sensory attributes of the muffin sample. Abbreviations used: M0%: control formulation without WL (100% sunflower oil). MV20%, MV35%, MV50%: formulations in which 20%, 35% or 50% of sunflower oil was replaced by WL originating from Viorica. MFR20%, MFR35%, MFR50%: formulations in which 20%, 35% or 50% of sunflower oil was replaced by WL originating from Fetească Regală.

The sensory evaluation was performed by a panel of trained assessors who scored the samples on a 5-point scale. Based on the average scores for seven different formulations produced with WL from the Viorica and Fetească Regală grape varieties, a radar plot was constructed to visualize the sensory profiles (Figure 5). The statistical analysis of sensory scores confirmed significant differences among muffin formulations for several evaluated attributes (*p* < 0.05), supporting the observed changes in sensory profile with increasing WL incorporation.

The addition of WL affected nearly all aroma- and taste-related attributes. The WL substitution led to a reduction in perceived egg taste and enhanced wine-derived aroma notes. Many panelists described honey-like, grape, and raisin notes in the enriched samples, consistent with the aromatic profile of the WL used. The samples with 20% and 35% fat replacement received the most favorable flavor and aroma scores, surpassing even the control. The MFR35% muffin achieved the highest aroma (4.95) and taste (4.85) ratings, compared with 4.80 and 4.70 for the control, respectively, indicating a positive contribution of WL to the sensory profile. Furthermore, samples prepared with WL from Fetească Regală were rated 1.40% higher overall than those produced with WL from Viorica.

Color intensity increased proportionally with the level of WL added. Muffins enriched with higher amounts of WL exhibited a darker appearance, with the most pronounced color observed in sample MFR50% (Figure 4b). This darker tone is likely associated with Maillard browning reactions enhanced by the presence of WL components and is consistent with observations in bakery products fortified with white grape pomace [48]. Despite the darkening, panelists reported that the overall visual appeal remained acceptable and appetizing across all samples.

Textural attributes were also influenced by WL addition. Increasing the amount of WL led to firmer textures, with reduced porosity and slightly sticky crumb. Samples containing 50% WL substitution showed the lowest consistency score (3.97), reflecting the higher hardness noted by assessors. This trend can be explained by the high water-binding capacity of β-glucans present in the WL, which may reduce gluten network formation and lead to a denser, more compact structure [49].

Based on the overall assessment, the highest overall acceptability level was recorded for samples with a medium WL level. The MFR20% sample ranked first with 4.89 points, followed by MV20% with 4.85 points. These results indicate that muffins enriched with 20% WL can be highly valued by consumers and represent a competitive baked good with improved nutritional properties.

Substitution levels above 35% markedly altered the sensory characteristics of the muffins, leading to noticeable changes in aroma intensity, crumb structure, and overall mouthfeel. The 50% replacement samples exhibited increased hardness, reduced porosity, and a less uniform internal structure, accompanied by a weaker balance of flavor attributes. These alterations were consistently perceived by panelists as less desirable compared with samples containing moderate amounts of WL.

Differences in the sensory behavior of the muffins prepared with WL from Viorica and Fetească Regală were also evident. Samples containing lees from Fetească Regală generally exhibited more pronounced aromatic notes, a slightly sweeter perception, and a softer crumb in comparison with those enriched with lees from Viorica. These distinctions can be attributed to the higher carbohydrate content in the FR sediment, compared with the lower carbohydrate levels characteristic of the Viorica sediment. The increased carbohydrate fraction in FR lees may enhance Maillard reactions, volatile compound formation, and water-binding interactions during baking, thereby directly influencing their organoleptic properties. Consequently, the FR-based formulations tended to deliver a more balanced flavor profile and improved sensory acceptability, while the Viorica-based samples produced a slightly denser texture and more restrained aromatic expression.

### 3.6. Microbiological Analysis

The microbiological quality of the muffin samples enriched with WL was assessed by determining the total viable count (TVC), the presence of coliform bacteria, coagulase-positive *Staphylococcus, Salmonella* spp., mold fungi, and yeast cells. The results are summarized in Table 5.

The incorporation of WL had a notable impact on the microbiological profile of the muffins. The control sample recorded the highest TVC value (5.70 × 10^2^ CFU/g), while a progressive reduction was observed with increasing levels of WL. In muffins enriched with Viorica lees, TVC decreased from 2.71 × 10^2^ CFU/g at 20% replacement to 1.33 × 10^2^ CFU/g at 50%. A similar trend was recorded in muffins containing Fetească Regală lees, where TVC declined from 3.10 × 10^2^ CFU/g (20%) to 2.77 × 10^2^ CFU/g (50%). These reductions demonstrate a dose-dependent inhibitory effect of WL on total viable microorganisms.

No coliforms, coagulase-positive staphylococci, *Salmonella* spp., or mold fungi were detected in any of the samples, indicating satisfactory hygienic conditions and effective thermal treatment during baking. Yeast counts, however, increased proportionally with the degree of replacement: from 3 ± 0 CFU/g in the control sample to 48 ± 0 and 71 ± 2 CFU/g at 20% and 35% Viorica substitution, respectively, reaching 92 ± 0 CFU/g at 50%. A similar incremental pattern was observed for Fetească Regală formulations, with yeast counts rising from 45 ± 1 CFU/g (20%) to 68 ± 1 CFU/g (35%) and 95 ± 0 CFU/g (50%). This increase is attributable to the viable *Saccharomyces cerevisiae* cells naturally present in the untreated WL, which remain partially active after the baking stage. Their persistence in the final product could potentially influence microbiological stability during storage and may shorten shelf life under favorable conditions; however, this aspect was not directly assessed in the present study.

A similar microbiological behavior has been reported in other studies that incorporated winery by-products or natural ingredients into meat and bakery systems. The TVC values in our muffins (10^2^ CFU/g order of magnitude for all formulations) are comparable to those described for muffins preserved with natural plant ingredients, where total aerobic mesophilic counts remained around 1.7–2.2 log CFU/g and yeast–mould counts around 1.1–2.3 log CFU/g during storage, with *Salmonella* and *E. coli* consistently absent [50].

## 4. Conclusions

The results suggest that WL may act as a sustainable partial fat replacer in muffin formulations under the tested conditions, enabling lipid reduction while contributing to the nutritional profile of the final products. By partially substituting vegetable fat with WL derived from Viorica and Feteasca Regală grape varieties at levels of 20%, 35%, and 50%, it was possible to obtain muffins with reduced caloric content (up to 10%) and enhanced nutritional value due to increased levels of dietary fiber and protein. The experimental samples exhibited acceptable physical parameters, including dry matter content, titratable acidity, density, and water absorption capacity, all falling within normative limits. Chromatic analysis revealed that a 50% fat replacement led to perceptible color changes (∆H* = 19.4 and ∆E = 26.89 for MFR50%), which may negatively affect consumer perception. However, sensory evaluation confirmed high consumer acceptability for samples with 20% fat replacement (MFR20% and MV20%), which were described as having pleasant honey and dried fruit notes. These findings suggest that differences in carbohydrate composition between the two WL types may have influenced the organoleptic properties, with Feteasca Regală contributing to a richer sensory profile. Overall, WL presents a promising sustainable ingredient for healthier bakery formulations.

This study has several limitations. The results are limited to wine lees obtained from two local Moldovan grape varieties, Viorica and Fetească Regală, and to the specific substitution levels and processing conditions tested. In addition, the wine lees were evaluated mainly in terms of basic physicochemical and nutritional characteristics; therefore, further studies should investigate the effect of different pre-treatment or stabilization methods on their technological functionality and microbiological safety.

## Figures and Tables

**Table 1:** Experimental design of muffin formulations with WL as fat replacer.

Sunflower Oil Replaced Level, %	Wheat Flour, g	Sugar, g	Potable Water, g	Fresh Eggs, g	Sunflower Oil, g	WL *, g	Baking Powder, g	Salt, g
0%	100	50	50	35	25.00	0.00	2	1
20%	100	50	50	35	20.00	5.00	2	1
35%	100	50	50	35	16.25	8.75	2	1
50%	100	50	50	35	12.50	12.50	2	1

* WL—wine lees.

**Table 2:** Physicochemical and nutritional parameters of white WL.

Nr.	Parameters	White WL	
		Viorica	Feteasca Regala
1	pH	3.21 ± 0.11	3.19 ± 0.03
2	Ash, %	0.03 ± 0.15	0.04 ± 0.31
3	Dry matter, %	24.06 ± 0.12	25.69 ± 0.6
4	Carbohydrates, % DM	14.21 ± 0.74	19.41 ± 0.13
5	Lipids, % DM	11.09 ± 0.79	10.08 ± 0.13
6	Proteins, % DM	31.21 ± 0.35	45.11 ± 0.81
7	β-glucans, %	19.11 ± 0.04	21.75 ± 0.03
	Fractional composition of lipids in WL (% of total lipids ± standard deviation)		
8	Phospholipids	12.01 ± 0.05	11.01 ± 0.06
9	Sterols	3.80 ± 0.03	6.85 ± 0.13
10	Monoglycerides	7.10 ± 0.06	6.98 ± 0.06
11	Diglycerides	10.16 ± 0.05	8.23 ± 0.16
12	Triglycerides	58.02 ± 0.02	59.81 ± 0.05
13	Esters	8.91 ± 0.01	7.22 ± 0.03

Abbreviations used: DM—dry matter.

**Table 3:** Approximate nutritional value of muffins with added WL.

Sample	Ingredients	Quantity, g	Protein, g	Carbohydrates, g	Lipids, g	Dietary Fiber, g/100 g	Energy, kJ/100 g	Energy, kcal/100 g
	*Control sample*							
M 0%	Sunflower oil	9.50	0.00	0.00	9.50	0.00	357.31	85.40
	Wheat flour (HQ)	38.00	4.10	26.60	0.50	1.03	530.94	126.90
	Granulated sugar	19.03	0.00	19.00	0.00	0.00	317.56	75.90
	Drinking water	19.03	0.00	0.00	0.00	0.00	0.00	0.00
	Chicken eggs	13.30	1.70	0.10	1.60	0.00	89.11	21.30
	Baking powder	0.76	0.00	0.10	0.00	0.00	2.51	0.60
	Table salt	0.38	0.00	0.00	0.00	0.00	0.00	0.00
	Total M 0%	100.00	5.80	54.80	11.60	1.03	1297.43	310.10
	*Viorica*							
MMV 20%	WL *	1.90	1.48	0.30	0.07	0.36	32.42	7.75
	Sunflower oil	7.60	0.00	0.00	7.60	0.00	285.76	68.30
	Wheat flour (HQ)	38.00	4.10	26.60	0.50	1.03	530.94	126.90
	Granulated sugar	19.03	0.00	19.00	0.00	0.00	317.56	75.90
	Drinking water	19.03	0.00	0.00	0.00	0.00	0.00	0.00
	Chicken eggs	13.30	1.70	0.10	1.60	0.00	89.11	21.30
	Baking powder	0.76	0.00	0.10	0.00	0.00	2.51	0.60
	Table salt	0.38	0.00	0.00	0.00	0.00	0.00	0.00
	Total MV 20%	100.00	7.28	46.10	9.77	1.39	1258.30	308.85
MV 35%	WL *	3.33	2.60	0.52	0.12	0.63	56.73	13.56
	Sunflower oil	6.17	0.00	0.00	6.17	0.00	232.21	55.50
	Wheat flour (HQ)	38.00	4.10	26.60	0.50	1.03	530.94	126.90
	Granulated sugar	19.03	0.00	19.00	0.00	0.00	317.56	75.90
	Drinking water	19.03	0.00	0.00	0.00	0.00	0.00	0.00
	Chicken eggs	13.30	1.70	0.10	1.60	0.00	89.11	21.30
	Baking powder	0.76	0.00	0.10	0.00	0.00	2.51	0.60
	Table salt	0.38	0.00	0.00	0.00	0.00	0.00	0.00
	Total MV 35%	100.00	8.40	46.32	8.39	1.66	12,229.06	293.76
MV 50%	WL *	4.75	3.70	0.74	0.17	0.91	80.70	19.29
	Sunflower oil	4.75	0.00	0.00	4.75	0.00	178.65	42.70
	Wheat flour (HQ)	38.00	4.10	26.60	0.50	1.03	530.94	126.90
	Granulated sugar	19.03	0.00	19.00	0.00	0.00	317.56	75.90
	Drinking water	19.03	0.00	0.00	0.00	0.00	0.00	0.00
	Chicken eggs	13,3	1.70	0.10	1.60	0.00	89.11	21.30
	Baking powder	0.76	0.00	0.10	0.00	0.00	2.51	0.60
	Table salt	0.38	0.00	0.00	0.00	0.00	0.00	0.00
	Total MV 50%	100.00	9.50	46.54	7.02	1.94	1199.47	286.69
	*Feteasca Regală*							
MFR 20%	WL *	1.90	1.44	0.27	0.09	0.41	32.01	7.65
	Sunflower oil	7.60	0.00	0.00	7.60	0.00	285.76	68.30
	Wheat flour (HQ)	38.00	4.10	26.60	0.50	1.03	530.94	126.90
	Granulated sugar	19.03	0.00	19.00	0.00	0.00	317.56	75.90
	Drinking water	19.03	0.00	0.00	0.00	0.00	0.00	0.00
	Chicken eggs	13.30	1.70	0.10	1.60	0.00	89.11	21.30
	Baking powder	0.76	0.00	0.10	0.00	0.00	2.51	0.60
	Table salt	0.38	0.00	0.00	0.00	0.00	0.00	0.00
	Total MFR 20%	100.00	7.24	46.07	9.79	1.44	1257.89	300.65
MFR 35%	WL *	3.33	2.54	0.47	0.16	0.73	56.40	13.48
	Sunflower oil	6.17	0.00	0.00	6.17	0.00	232.21	55.50
	Wheat flour (HQ)	38.00	4.10	26.60	0.50	1.03	530.94	126.90
	Granulated sugar	19.03	0.00	19.00	0.00	0.00	317.56	75.90
	Drinking water	19.03	0.00	0.00	0.00	0.00	0.00	0.00
	Chicken eggs	13.30	1.70	0.10	1.60	0.00	89.11	21.30
	Baking powder	0.76	0.00	0.10	0.00	0.00	2.51	0.60
	Table salt	0.38	0.00	0.00	0.00	0.00	0.00	0.00
	Total MFR 35%	100.00	8.34	46.27	8.43	1.76	1228.73	293.68
MFR 50%	WL *	4.75	3.62	0.67	0.22	1.03	80.08	19.14
	Sunflower oil	4.75	0.00	0.00	4.75	0.00	178.65	42.70
	Wheat flour (HQ)	38.00	4.10	26.60	0.50	1.03	530.94	126.90
	Granulated sugar	19.03	0.00	19.00	0.00	0.00	317.56	75.90
	Drinking water	19.03	0.00	0.00	0.00	0.00	0.00	0.00
	Chicken eggs	13.30	1.70	0.10	1.60	0.00	89.11	21.30
	Baking powder	0.76	0.00	0.10	0.00	0.00	2.51	0.60
	Table salt	0.38	0.00	0.00	0.00	0.00	0.00	0.00
	Total MFR 50%	100.00	9.42	46.47	7.07	2.06	1198.85	286.54

Abbreviations used: M0%: control formulation without WL (100% sunflower oil). MV20%, MV35%, MV50%: formulations in which 20%, 35% or 50% of sunflower oil was replaced by WL originating from Viorica. MFR20%, MFR35%, MFR50%: formulations in which 20%, 35% or 50% of sunflower oil was replaced by WL originating from Fetească Regală. * the WL composition was experimentally determined as part of this study.

**Table 4:** Chromatic Parameters of WL and Muffins with WL.

Sample	L* (WL)	a* (WL)	b* (WL)	WI	L* (Crust)	L* (Crumb)	a* (Crust)	a* (Crumb)	b* (Crust)	b* (Crumb)	ΔH*	ΔE*
WL Viorica	18.36 ± 0.80	9.44 ± 0.92	10.77 ± 1.16	17.11 ± 0.80	–	–	–	–	–	–	–	–
WL Fetească Regală	29.22 ± 0.93	6.16 ± 0.94	12.64 ± 0.50	27.84 ± 0.92	–	–	–	–	–	–	–	–
M0%	–	–	–	–	54.42 ± 0.38	56.77 ± 0.95	10.52 ± 0.21	−3.39 ± 1.90	34.73 ± 0.79	25.06 ± 0.52	–	–
MV20%	–	–	–	–	53.20 ± 0.87	46.22 ± 0.85	17.47 ± 0.39	1.46 ± 0.04	47.10 ± 0.95	25.19 ± 0.85	4.85	11.61
MV35%	–	–	–	–	46.23 ± 0.80	45.15 ± 1.04	18.58 ± 0.18	3.94 ± 1.12	39.57 ± 0.82	18.71 ± 0.49	7.48	15.13
MV50%	–	–	–	–	41.80 ± 0.97	41.80 ± 1.01	21.02 ± 0.91	6.42 ± 0.53	38.56 ± 0.90	17.83 ± 0.71	8.16	16.86
MFR20%	–	–	–	–	44.39 ± 0.86	50.79 ± 1.01	16.37 ± 0.61	−2.82 ± 0.75	27.95 ± 0.68	15.96 ± 0.86	8.06	13.44
MFR35%	–	–	–	–	23.44 ± 1.01	51.90 ± 0.95	17.83 ± 0.97	−2.71 ± 1.30	26.61 ± 1.00	13.48 ± 1.08	10.06	16.97
MFR50%	–	–	–	–	13.99 ± 0.85	47.32 ± 0.98	18.83 ± 0.93	3.21 ± 0.70	12.48 ± 1.08	12.49 ± 0.97	19.40	26.89

Note: Results indicate the mean value of three independent assays and are expressed as mean ± standard deviation (SD); Abbreviations used: M0%: control formulation without WL (100% sunflower oil). MV20%, MV35%, MV50%: formulations in which 20%, 35% or 50% of sunflower oil was replaced by WL originating from Viorica. MFR20%, MFR35%, MFR50%: formulations in which 20%, 35% or 50% of sunflower oil was replaced by WL originating from Fetească Regală; WI—whiteness index.

**Table 5:** Microbiological indicators of muffins with WL.

Sample Code	Total Viable Count (TVC), CFU/g	Coliform Bacteria	Coagulase-Positive Staphylococci	Pathogenic Microorganisms (e.g., *Salmonella* spp.)	Mold Fungi, CFU/g	Yeast, CFU/g
M (0%)	(5.70 ± 0.08) × 10^2^	nd	nd	nd	nd	3 ± 0
MV 20%	(2.71 ± 0.03) × 10^2^	nd	nd	nd	nd	48 ± 0
MV 35%	(2.54 ± 0.01) × 10^2^	nd	nd	nd	nd	71 ± 2
MV 50%	(1.33 ± 0.09) × 10^2^	nd	nd	nd	nd	92 ± 0
MFR 20%	(3.10 ± 0.02) × 10^2^	nd	nd	nd	nd	45 ± 1
MFR 35%	(2.80 ± 0.05) × 10^2^	nd	nd	nd	nd	68 ± 1
MFR 50%	(2.77 ± 0.04) × 10^2^	nd	nd	nd	nd	95 ± 0

Note: nd = not detected. Abbreviations used: M0%: control formulation without WL (100% sunflower oil). MV20%, MV35%, MV50%: formulations in which 20%, 35% or 50% of sunflower oil was replaced by WL originating from Viorica. MFR20%, MFR35%, MFR50%: formulations in which 20%, 35% or 50% of sunflower oil was replaced by WL originating from Fetească Regală.

## Data Availability

The original contributions presented in this study are included in the article. Further inquiries can be directed to the corresponding author.
